# Supplementary material for: Comparison between Adult Patients with Sickle Cell Disease of Sub-Saharan African Origin Born in Metropolitan France and in Sub-Saharan Africa
Source: J Clin Med. 2019 Dec 9;8(12):2173. doi: 10.3390/jcm8122173 (PMC6947353; doi:10.3390/jcm8122173)
Supplement: Supplementary file 1 [file jcm-08-02173-s001.pdf]

Table S1. Comparison of main characteristics, acute and chronic complications and therapeutic management in SS/Sβ0 patients according to the region of birth.

	**SS/Sβ0: Whole Population *n* = 186**	**SS/Sβ0: Patients Born in France *n* = 93**	**SS/Sβ0: Patients Born in Sub–Saharan Africa *n* = 93**	***p*** [a]	***p*** [b]
**Male**, *n* (%)	91 (48.9)	50 (53.8)	41 (44.1)	0.2	0.5
**Age at last follow–up** (years)	27.9 (22.4–34.1)	25.6 (22.2–30.2)	30 (23–36.8)	**0.001**	**<0.0001**
**Height** (cm)	172 (165–178)	174.3 (167.8–178.6)	170 (163–177.8)	**0.01**	**0.007**
Male (cm)	177 (172–182)	176 (173.6–182)	178 (171.8–180.3)	0.8	0.4
Female (cm)	167 (161–171)	168.8 (164.3–173.8)	164 (160–169.5)	**0.009**	**0.004**
**Weight** (kg)	63 (57.1–70)	63.5 (59–72)	63 (56.5–70)	0.1	0.01
**BMI** (kg/m^2)	21.3 (19.8–23.8)	21.2 (19.8–24.2)	21.5 (19.9–23.7)	0.9	0.1
**Hb** (g/dL)	8.4 (7.5–9.4)	8.2 (7.5–9.2)	8.5 (7.9–9.6)	0.1	0.2
**MCV** (fL)	82 (75.3–89.8)	80 (75–89)	82.2 (76–91.4)	0.4	1
**Reticulocytes** (G/L)	267 (182.3–347.3)	256 (169.5–336)	275 (184.5–347.8)	0.6	0.2
**LDH** (UI/L)	438 (361.8–516.8)	438 (325–531.5)	438 (386.5–503)	0.6	0.2
**Total bilirubin** (µmol/L)	42 (29–75)	39 (29–77.5)	48 (27.5–66)	0.6	0.7
**HbA2** (%)	3.3 (3–3.7)	3.4 (3–3.5)	3.3 (2.9–3.7)	1	0.5
**HbF** (%)	5.5 (2.8–8.5)	5.1 (2–8.5)	6.7 (3.2–8.8)	0.2	0.3
**Vaso–occlusive crisis**					
Number of admissions for VOC in the last 12 months	1 (0–2)	1 (0–3)	1 (0–2)	0.1	0.9
**Acute chest syndrome**, *n* (%)	145/185 (78.4)	80/93 (86)	65/92 (70.7)	**0.02**	**0.005**
Number of episodes over lifetime	2 (1–4)	3 (1–4)	1 (0–3)	**<0.0001**	**0.002**
Age at first episode (years)	20.9 (15.7–24.7)	19.2 (11.6–22.3)	23.5 (18.3–28.9)	**<0.0001**	**0.01**
≥1 ICU admission for ACS over lifetime	88/174 (50.6)	53/90 (58.9)	35/84 (41.7)	**0.03**	**0.008**
**Priapism**, *n* (%)	31/81 (38.3)	17/45 (37.8)	14/36 (38.9)	1	0.7
Age at 1st priapism episode (years)	16.8 (12.6–22.4)	18 (12.9–22.8)	15.3 (12.2–18.2)	0.5	0.7
**Stroke,** *n* (%)	17/177 (9.6)	6/90 (6.7)	11/87 (12.5)	0.3	0.3
Age at first stroke (years)	19 (11.1–23.3)	17.1 (12.9–21.2)	19 (11–28.4)	0.8	0.1
**Splenic complications**					
Splenectomy, *n* (%)	15/175 (8.6)	8/87 (9.2)	7/88 (8)	1	0.6

Age at splenectomy (years)	11.3 (9–14.8)	12.1 (7.6–15.4)	10.9 (9.5–13.5)	0.9	0.2
**Cholecystectomy,** *n* (%)	121/181 (66.9)	68/92 (73.9)	53/89 (59.6)	0.06	**0.04**
Age at cholecystectomy (years)	16.8 (12.5–23.3)	14.4 (10.9–20.2)	21.1 (14.8–28.3)	**<0.0001**	1
**Thrombo–embolic events**, *n* (%)	14/183 (7.7)	8/91 (8.8)	6/92 (6.5)	0.8	0.2
Pulmonary embolism, *n* (%)	11/183 (6)	6/91 (6.6)	5/92 (5.4)	1	0.4
Age at 1st thrombo–embolic event (years)	27.3 (24–30.3)	28.4 (23.2–30.6)	26.1 (24.6–28.3)	0.7	0.2
**Retinopathy**, *n* (%)	72/163 (44.2)	39/83 (47)	33/80 (41.3)	0.6	0.2
Age at diagnosis of retinopathy (years)	23.2 (20.3–29.3)	21.2 (16.7–25.8)	27.8 (23–30.9)	**<0.0001**	0.2
Laser photocoagulation, *n* (%)	29/146 (19.9)	18/76 (23.7)	11/70 (15.7)	0.3	0.05
**Cardiac involvement**, *n* (%)	64/146 (43.8)	34/74 (46)	30/72 (41.7)	0.7	0.7
Age at diagnosis of cardiopathy (years)	25.7 (20.5–29.7)	24.5 (20.5–28.6)	27.5 (21–30.6)	0.2	0.7
LV systolic dysfunction, *n* (%)	14/181 (7.7)	8/91 (8.8)	6/90 (6.7)	0.8	0.2
LV and/or LA dilatation, *n* (%)	53/136 (39)	29/72 (40.3)	24/64 (37.5)	0.9	0.6
Age at diagnosis of LV dilatation (years)	25.1 (20.2–29.2)	24.5 (18.2–28.4)	27.4 (21.1–30.4)	0.08	0.5
TRV ≥ 2.5 m/s, *n* (%)	14/161 (8.7)	6/86 (7)	8/75 (10.7)	0.6	0.8
**Cerebral vasculopathy**, *n* (%)	32/96 (33.3)	13/50 (26)	19/46 (41.3)	0.2	0.2
Brain aneurysms, *n* (%)	13/92 (14.1)	4/50 (8)	9/42 (21.4)	0.1	0.09
Silent cerebral infarcts, *n* (%)	10/79 (12.7)	6/43 (14)	4/36 (11.1)	0.7	0.4
Moyamoya, *n* (%)	2/91 (2.2)	1/53 (1.9)	1/38 (2.6)	1	0.9
Vessel stenosis, *n* (%)	7/91 (7.7)	2/53 (3.8)	5/38 (13.2)	0.8	0.7
**Nephropathy**, *n* (%)	60/129 (46.5)	29/70 (41.4)	31/59 (52.5)	0.3	0.4
eGFR (mL/mn/1.73 m^2)	126 (112.1–136.2)	128.2 (120.2–137.4)	121.9 (105.2–134.6)	**0.009**	0.07
Hyperfiltration [§], *n* (%)	29/176 (16.5)	14/89 (15.7)	15/87 (17.2)	0.9	0.4
Chronic kidney insufficiency [¶], *n* (%)	4/174 (2.3)	0/89 (0)	4/85 (4.7)	0.06	1
ACR > 3 mg/mmol, *n* (%)	50/114 (43.9)	25/62 (40.3)	25/52 (48.1)	0.5	0.6
ACR (mg/mmol)	2 (0.9–9.3)	1.7 (0.8–6.8)	2.3 (1.3–12.2)	0.1	0.4
**Bone complications**					
Avascular osteonecrosis (AON), *n* (%)	60/172 (34.9)	32/89 (36)	28/83 (33.7)	0.9	0.3
Age at diagnosis of AON (years)	18.9 (16.6–26.6)	18.9 (16.6–25.2)	18.9 (16.5–28.6)	0.6	0.3
H–shaped vertebrae, *n* (%)	39/76 (51.3)	18/38 (47.4)	21/38 (55.3)	0.6	0.5
Fracture, *n* (%)	48/145 (33.1)	29/72 (40.3)	19/73 (26)	0.1	0.05

Osteomyelitis, *n* (%)	38/169 (22.5)	23/80 (28.8)	15/89 (16.9)	0.1	**0.04**
Age at 1st osteomyelitis episode (years)	10.8 (6.4–19.2)	7.7 (2.8–19.2)	12.1 (8.9–18.8)	0.1	1
**Skin ulcers**, *n* (%)	22/151 (14.6)	6/80 (7.5)	16/71 (22.5)	**0.01**	**0.04**
Age at first episode (years)	18.6 (16.8–27.5)	23.2 (18.6–29.6)	18.5 (14.4–25.8)	0.2	**0.005**
**Hydroxyurea**					
Lifetime exposure, *n* (%)	131/186 (70.4)	69/93 (74.2)	62/93 (66.7)	0.3	0.2
Current treatment, *n* (%)	105/186 (56.5)	54/93 (58.1)	51/93 (54.8)	0.8	0.7
Age at introduction (years)	19.4 (15.4–24.8)	18.5 (14.3–23.2)	22.1 (16.4–28.5)	**0.003**	**0.002**
Cumulative lifetime dose (g)	1396 (502.5–2470.8)	1299.3 (540.3–2748.2)	1424.5 (456–1963)	0.7	0.2
Cumulative lifetime duration (years)	4 (1.6–8.1)	3.9 (1.9–8)	4.2 (1.4–7.9)	0.8	**0.003**
**Chronic blood transfusion program**					
Previous, *n* (%)	60/177 (33.9)	28/89 (31.5)	32/88 (36.4)	0.6	0.9
Current, *n* (%)	28/180 (15.6)	17/93 (18.3)	11/87 (12.6)	0.4	0.2
**Blood transfusion complications**, *n* (%)	41/154 (26.6)	22/80 (27.5)	19/74 (25.7)	0.9	0.6
Types of transfusion complications *:					
Antibodies without hemolytic reaction, *n* (%)	31/40 (77.5)	17/22 (77.3)	14/18 (77.8)	1	0.7
DHTR with antibodies, *n* (%)	6/40 (15)	4/22 (18.2)	2/18 (11.1)	0.7	0.7
DHTR without antibodies, *n* (%)	2/40 (5)	1/22 (4.6)	1/18 (5.6)	1	0.5
Acute hemolytic transfusion reaction, *n* (%)	1/40 (2.5)	0/22 (0)	1/18 (5.6)	0.5	1

SCD: sickle cell disease, BMI: body mass index, Hb: hemoglobin, MCV: mean corpuscular volume, LDH: lactate dehydrogenase, HbA2: adult hemoglobin A2, HbF: fetal hemoglobin. VOC = vaso-occlusive crisis, ACS = acute chest syndrome, LV: left ventricle, LA: left atrial, TRV: tricuspid regurgitation velocity, ACR: urine albumin-to-creatinine ratio, AON: avascular osteonecrosis, eGFR: estimated glomerular filtration rate. § Renal hyperfiltration was defined as an eGFR ≥130 mL/min/1.73m^2 for women and ≥140 mL/min/1.73 m^2 for men. ¶ Chronic kidney insufficiency was defined by an eGFR < 60 mL/min/1.73 m^2. DHTR: delayed hemolytic transfusion reactions, MRI: magnetic resonance imaging. Continuous variables are expressed as the median [IQR]. [a] bivariate analysis: Chi^2, Fisher's, Student's or Wilcoxon test. [b] multivariate analysis with adjustment for age, gender and genotype. Significant p values appear in bold.
